# Use of intravascular ultrasound for optimal vessel sizing in chronic total occlusion percutaneous coronary intervention

**DOI:** 10.3389/fcvm.2022.922366

**Published:** 2022-08-03

**Authors:** Recha Blessing, Andrea Buono, Majid Ahoopai, Martin Geyer, Maike Knorr, Moritz Brandt, Sebastian Steven, Ioannis Drosos, Thomas Muenzel, Philip Wenzel, Tommaso Gori, Zisis Dimitriadis

**Affiliations:** ^1^University Medical Center Mainz, Center of Cardiology, Johannes Gutenberg University, Mainz, Germany; ^2^Interventional Cardiology Unit, Fondazione Poliambulanza, Brescia, Italy; ^3^Center for Thrombosis and Hemostasis, Johannes Gutenberg University, Mainz, Germany; ^4^German Center for Cardiovascular Research (DZHK), Mainz Partner Site Rhine-Main, Mainz, Germany; ^5^Division of Cardiology, Department of Medicine III, University Hospital Frankfurt, Goethe University Frankfurt am Main, Frankfurt, Germany

**Keywords:** intravascular ultrasound (IVUS), percutaneous coronary intervention (PCI), CTO percutaneous coronary intervention, coronary artery disease, complex PCI

## Abstract

**Aim:**

The aim of this study is to provide evidence on how use of standardized intravascular ultrasound (IVUS) use impacts stent size choice in the setting of chronic total occlusion (CTO) percutaneous coronary intervention (PCI) compared to visual estimation.

**Methods and results:**

Data of 82 consecutive patients who had successfully undergone IVUS-guided revascularization of CTO at the University Medical Center Mainz were analyzed. Angiography-based stent size prediction for the proximal and distal vessels was compared to the implanted stent diameter after IVUS assessment. Angiography-based stent size prediction for the proximal vessel was 3.09 ± 0.41, whereas IVUS use demonstrated larger vessel diameter, resulting in larger implanted stent diameter (3.24 ± 0.45, *p* < 0.001). Proximal vessel stent size prediction was underestimated in the majority of patients by angiographic estimation. Angiography-based stent size prediction for the distal vessel was 2.79 ± 0.38, whereas IVUS use demonstrated larger vessel diameter, resulting in larger implanted stent diameter (2.92 ± 0.39, *p* < 0.001).

**Conclusion:**

Pre-stent IVUS assessment in CTO PCI provides important information on vessel morphology and size. Angiography-based stent size prediction for the proximal and distal vessels was frequently underestimated, IVUS use demonstrated larger vessel diameter, resulting in significantly larger implanted stent diameter.

## Introduction

Revascularization of a chronic total occlusion (CTO) of a coronary artery considered as a complex percutaneous coronary intervention (PCI), with higher rates of procedural failure (1), complications (2, 3), and in-stent restenosis than less complex PCIs (4, 5). Advances in catheter techniques, materials, and treatment algorithms have increased the success rate of CTO PCI.

In addition to these technical advances, intracoronary imaging, particularly intravascular ultrasound (IVUS), has been proposed as a tool to optimize CTO recanalization procedures (1, 6, 7).

Currently, the use of IVUS is recommended for positioning and crossing of the guidewire with the Global Chronic Total Occlusion Crossing Algorithm (e.g., for penetrating of the cap, confirming true lumen positioning after antegrade dissection and re-entry, ADR, and controlled antegrade and retrograde tracking, CART) and increases the safety and efficiency of CTO PCIs (6–10).

Besides information for wire placement, IVUS also provides information about lesion length, morphology, and vessel diameter (11), allowing for optimization of stent selection, expansion, and apposition (12–14). The correct choice of stent length and diameter is a mandatory step to avoid strut malapposition due to undersizing and incomplete coverage of the lesion. In fact, in the CTO scenario, correct stent choice by visual/angiographic assessment is challenging even for experienced operators, as the distal vessel is often narrow, diffusely diseased, and degenerated because of chronic hypoperfusion (15–17). Therefore CTO PCIs result in high occurrence of stent-vessel mismatch due to difficult visual estimation of vessel size in the CTO context.

The aim of this study was to investigate the difference between angiography- and IVUS-assessed vessel diameter in patients undergoing CTO PCI and to show that IVUS assessment is a mandatory step not only for guidewire positioning and post-stent control but also delivers important information before stent implantation.

## Materials and methods

### Study design and study population

The study was prospectively conducted from July 2019 to July 2021. Data from 82 consecutive patients (≥18 years) who had successfully undergone IVUS-guided revascularization of CTO at the University Medical Center Mainz were analyzed. CTO was defined as a lesion with 100% stenosis and Thrombolysis In Myocardial Infarction (TIMI) flow grade 0 that exists for more than 3 months. The duration of occlusion was determined either based on the clinical record of previous coronary angiograms or clinical (onset of symptoms) or angiographic probability (e.g., collateralization). In-stent CTOs were considered as an exclusion criterion.

Coronary angiography and subsequent PCI were performed by an experienced operator in the CTO field and intracoronary imaging. The CTO hybrid algorithm (18, 19) was used in all the cases, starting with antegrade approaches and, in case of failure, escalation in retrograde approach. IVUS studies were performed using a commercially available system (PHILIPS Volcano; Cambridge, MA, United States).

Once the coronary wire crossed the CTO body and reached the distal true lumen, by protocol, the entire diseased vessel was be predilatated with a 2-mm noncompliant (NC) balloon in order to allow perfusion and vessel diameter assessment. All estimations were conducted by the same 4 experienced interventional cardiologists who assessed all the 82 lesions.

**FIGURE 1:**
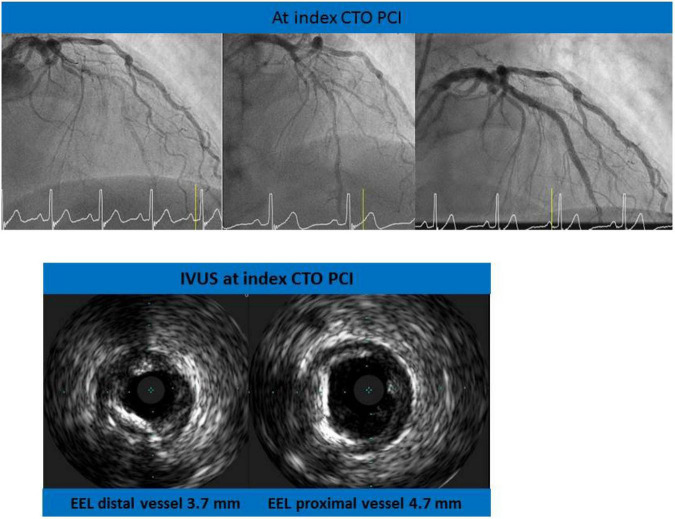
Revascularization of the left anterior descending artery (LAD). Examples of intravascular ultrasound (IVUS) assessment of the chronic total occlusion (CTO) vessel: distal and proximal vessel diameters.

The CTO operator (who performed the procedure) and the three experienced interventional cardiologists were asked to choose the size of the stent(s) on the basis of visual proximal and distal vessel diameter estimation. Predicted proximal and distal vessel diameters have been intended as the reference vessel diameters situated, respectively 5 mm proximally and distally to the CTO caps. Thereafter, IVUS assessment of the vessel was performed: distal and proximal vessel diameters (defined as mean diameter, an average of of minimal and maximal diameters) were calculated. External elastic lamina (EEL) to external elastic lamina was meassured by IVUS to assess the vessel diameter (Figure 1).

The size of the stents was selected on the basis of the mean diameter (with a 1:1 or near 1:1 ratio). If one stent was sufficient to treat the entire lesion, stent size was selected according the mean distal vessel diameter, and the proximal vessel diameter was used to determine the balloon diameter for proximal optimization. In cases with a relevant difference between distal and proximal mean diameters (>1 mm), an extra stent in the proximal part with a more suitable diameter was implanted in order to avoid stent fracture after a POT.

After stent implantation, post-dilatation was routinely performed with NC balloons in order to achieve a 1:1 ratio between stent and vessel diameter in all the treated segments. IVUS assessment was conducted on all the patients to determine stent length and achieve complete coverage of the lesion.

Second-generation drug-eluting stents were implanted in all the patients. Recommendations regarding antiplatelet regime after intervention were carried out in adherence to the guidelines (20). A follow-up with outpatient visit and surveillance angiography was performed after 6 months.

The study conformed to the Declaration of Helsinki and was approved by the local ethics committee. All the participants provided written informed consent.

### Statistical analysis

Normal distribution was tested by QQ-plot analysis and the Kolmogorov–Smirnov test. Continuous normally distributed data were presented as mean and standard deviation, and differences were tested by the Student‘s *t*-test; Non-normally distributed variables were presented as median and minimum and maximum values, and group comparisons were performed by the Mann-Whitney U test. Categorical data were presented as absolute and relative frequencies, and comparisons between groups were performed by chi -square test. Differences were considered statistically significant if *p* < 0.05.

The statistical analyses were performed using SPSS (version 23; IBM SPSS Statistics).

## Results

Eighty-two patients with successful IVUS-guided CTO PCI were prospectively included in the study. Clinical follow-up (outpatient visit and surveillance coronary angiography) was available for 72 (87.8%) of the patients. The mean follow-up period was 210 ± 20 days.

### Stent parameters

The average number of implanted stents was 2 ± 0.8. The estimated proximal and distal stent diameters of the operator were analyzed, and we found the following results for the proximal part of the lesion: angiography-based stent size prediction for the proximal vessel was 3.09 ± 0.41 mm, whereas IVUS use demonstrated larger vessel diameter, resulting in significantly larger implanted stent diameter (3.24 ± 0.45 mm, *p* < 0.001).

**FIGURE 2:**
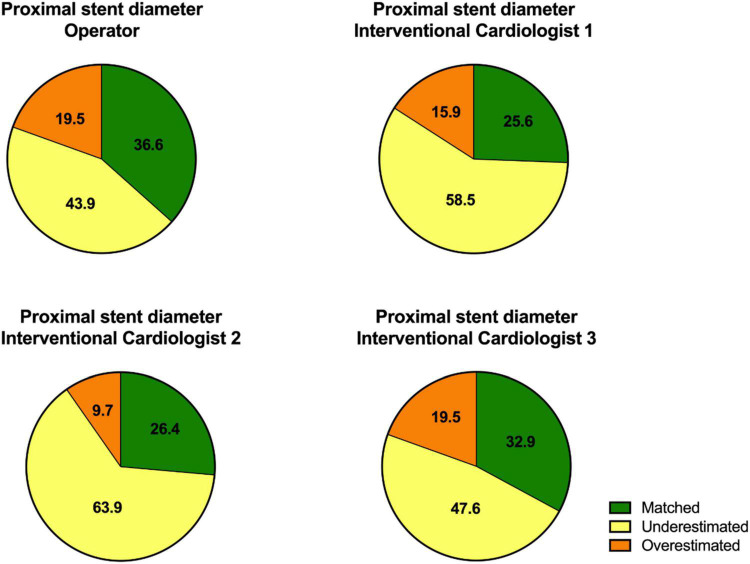
The diagrams show the values of the proximal stent diameter estimated by the operator who performed the CTO PCI and the three additional interventional cardiologists.

The analysis of the estimated proximal stent diameter by the other interventional cardiologists (interventional cardiologists 1, 2, and 3) also showed that proximal stent diameter was underestimated in majority of the patients by angiography. The results of the proximal stent parameters are shown in Figure 2 and Table 1.

**TABLE 1:** Proximal stent parameters [estimation, intravascular ultrasound (IVUS), and implantation].

IVUS guided PCI (*n* = 82)			

	Estimated proximal stent diameter	Implanted proximal stent diameter	*p*-value
Operator 1	3.09 ± 0.41 mm	3.24 ± 0.45 mm	**0.001**
Interventionalcardiologist 1	2.94 ± 0.45 mm		**<0.001**
Interventionalcardiologist 2	2,89 ± 0.29 mm		**<0.001**
Interventionalcardiologist 3	3.08 ± 0.54 mm		**0.004**
IVUS value	3.87 ± 0.64 mm		

Values are mean ± SD.

The analysis of the distal part of the lesion showed the following results: angiography-based stent size prediction for the distal vessel was 2.79 ± 0.38 mm, whereas IVUS use demonstrated larger vessel diameter, resulting in significantly larger implanted stent diameter (2.92 ± 0.39 mm, *p* < 0.001).

The analysis of the estimated distal stent diameter by the other interventional cardiologists (interventional cardiologists 1, 2, and 3) also showed that distal stent diameter was underestimated in majority of the patients by angiography.

**FIGURE 3:**
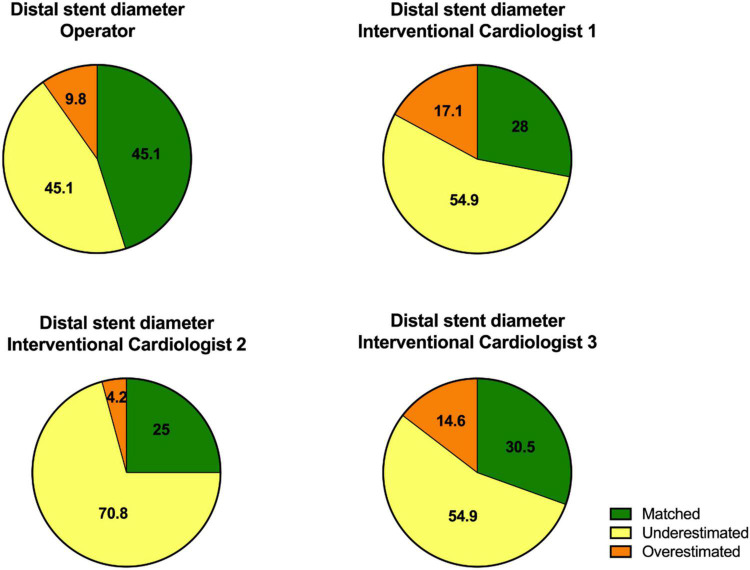
The diagrams show the values of the distal stent diameter estimated by the operator who performed the CTO PCI and the three additional interventional cardiologists.

The results of the distal stent parameters are presented in Figure 3 and Table 2.

**TABLE 2:** Distal stent parameters (estimation, IVUS, and implantation).

IVUS guided PCI (*n* = 82)			

	Estimated distal stent diameter	Implanted distal stent diameter	*p*-value
Operator 1	2.79 ± 0.38 mm	2.92 ± 0.39 mm	**<0.001**
Interventionalcardiologist 1	2.77 ± 0.39 mm		**<0.001**
Intervetnionalcardiologist 2	2.61 ± 0.26 mm		**<0.001**
Intervetnionalcardiologist 3	2,70 ± 0.49 mm		**<0.001**
IVUS value	3.15 ± 0.49 mm		

Values are mean ± SD.

### Clinical and angiographic outcomes

After discharge, none of the patients suffered from a cardiac event (cardiac death, nonfatal myocardial infarction, and stent thrombosis) within 6 months of the follow-up period. We observed 2 (2.77%) re-occlusions and 6 (8.33%) target lesion revascularizations on the 6-month surveillance coronary angiography. None of the patients developed acute renal failure after the CTO PCI. The comparison of GRF values before and 1 day after the CTO PCI showed no difference [81 (10–117) vs. 80.5 (14–120), p.15]. Clinical and angiographic parameters at baseline are shown in Table 3. Table 4 summarizes the clinical and angiographic outcomes at follow-up.

**TABLE 3:** Clinical and angiographic parameters at baseline.

	IVUS guided (*n* = 82)
**Demographics characteristics**	
Age, yrs	62.13 ± 11.20
Male	69 (84.1)
BMI kg/m^2^	27.40 (21.64–43.03)
Diabetes mellitus	16 (19.5)
Hypertension	70 (85.4)
Hyperlipidemia	77 (93.9)
Current smoking	21 (25.6)
Ex-smoker	24 (29.9)
Multivessel CAD	69 (84.1)
GFR (ml/min)	81 (10–117)
LVEF (%)	55 (15–66)
LVEF ≤ 40%	10 (12.3)
Previous stroke	5 (6.1)
PAD	11 (13.4)
Previous CABG	7 (8.5)
Previous MI	25 (30.5)
Previous PCI	59 (72)
**Procedural characteristics**	
CTO vessel	
RCA	41 (50.0)
LAD	19 (23.3)
LCX	22 (26.8)
J-CTO Score	1.78 ± 0.73
Antegrade access	79 (96.3)
Number of stents ≤ 3	3 (3.7)
Total stent length >20 mm	57 (69.5)
Fluoroscopic time (min)	25.65 (10.62–55.48)
Contrast (ml)	208 (48–450)
**Complications at baseline**	
Bleeding	0
Ventricular fibrillation	0
complication of access side	0
Stroke	0
Cardiac death	0
Acutekidneyfailure	0

Values are n (%), median (minimum-maximum), or mean ± SD.

yrs, years; BMI, body mass index; CAD, coronary artery disease; GFR, glomerular filtration rate; LVEF, left ventricular ejection fraction; PAD, peripheral artery disease; CABG, coronary artery bypass graft; MI, myocardial infarction; PCI, percutaneous coronary intervention; RCA, right coronary artery; LAD, left anterior descending coronary artery; and LCX, left circumflex coronary artery.

**TABLE 4:** Clinical and angiographic outcomes at follow-up.

Angiographic outcome	IVUS guided (*n* = 72)
Re-occlusion	2 (2.77)
TLR	6 (8.33)
Acute MI	0
Major bleeding	0

Values are n(%).

TLR, target lesión revascularization; and MI, myocardial infarction.

## Discussion

The main findings of our study are the following: first, values of the proximal and distal vessel diameters were estimated commonly smaller by visual assessment than by IVUS, which led to change in implanted stent diameter. Second, IVUS assessment was associated with good outcomes in the angiographic and clinical follow-up. Third, we found IVUS assessment to be safe and feasible in CTO PCI with low rate of complications.

Intravascular ultrasound-guided PCI is the most effective method to perform an optimal PCI with low rates of target lesion revascularization, target vessel revascularization, and major adverse cardiac events, but in the clinical routine, it is frequently underused. A meta-analysis published in 2016 showed that IVUS guided PCI reduces major adverse cardiac events (all-cause and cardiovascular deaths, myocardial infarction, target lesion revascularization, and target vessel revascularization) and stent thrombosis compared to angiography-guided PCI in complex lesions (21). To date, there are only a few studies investigating the effects and benefits of IVUS-guided CTO PCI.

Compared to the IVUS assessed diameter, the vessel diameter assessed by angiography was frequently underestimated in our collective. Estimation of vessel diameter by angiography is often complicated by significant calcification and tortuosity of the diffusely diseased and narrowed CTO vessel. In contrast, by IVUS, EEL to EEL is measured to assess vessel diameter, and this method is less affected by these factors. Another challenge of angiographic assessment was found by Allahwala et al. They showed in a collective of 174 patients that the distal vessel size was increased by 31.1% after successful CTO recanalization (17).

Kalogeropoulos et al. demonstrated in an observational study that after IVUS assessment of the lesion significantly longer stents and larger stent diameter were implanted in CTO PCI. In the clinical follow-up, there was no difference in the rate of clinical events (all-cause death, cardiac death, myocardial infarction, and target vessel revascularization) (22). Based on these data, IVUS offers the possibility for accurate measurement of vessel diameter and lesion length and enables optimal stent choice in the CTO PCI setting. An optimal stent choice (good stent apposition and complete lesion coverage) reduces the rate of restenosis and adverse clinical events (all-cause death, cardiac death, and myocardial infarction) after PCI (12, 23, 24).

In our collective, we found a significant difference between estimated and implanted stent diameters after IVUS use, which underscores the difficulty of assessment of stent diameter by angiography and the benefit of pre-stent IVUS use. Both proximal and distal stent diameters were underestimated.

Our collective showed a low restenosis rate in the follow-up, and this is most likely explained by the optimized choice of stent diameter by IVUS guidance.

Our study has several limitations. First, the sample size is limited and is not adequately powered to address the clinical endpoints. For this purpose, our analysis should be intended as hypothesis generating, and further prospective studies with larger cohorts are needed to investigate the benefit of pre-stent IVUS assessment in CTO PCI. Second, the IVUS technique was mandatorily used to choose the stent diameter, but we did not recommend by protocol a post-stent IVUS assessment. Moreover, information concerning the pre-recanalization status of the distal target vessel has not been routinely collected: the presence of CTOs with well-developed collateral could have mitigated IVUS usefulness in stent size choice, since angiographic estimation seems easier if the distal vessel is not diseased. Lastly, the follow-up was short, and this could explain the low number of major clinical events.

## Conclusion

Intravascular ultrasound is an important tool to achieve a procedural and short-term efficacy in the CTO scenario.

Based only on angiographic appearance, proximal and distal reference vessel diameters were often underestimated when compared to intravascular ultrasound assessment. This aspect has led to change in stent selection, with a low rate of TLR at 6-month follow-up.

## Data availability statement

The raw data supporting the conclusions of this article will be made available by the authors, without undue reservation.

## Ethics statement

The studies involving human participants were reviewed and approved by the Ethics Committee of Rhineland Palatinate. The patients/participants provided their written informed consent to participate in this study.

## Author contributions

ZD, AB, and TG: conceptualization and supervision. ZD and AB: methodology. RB: software, formal analysis, data curation, writing (original draft preparation), and visualization. ZD, TG, AB, and RB: validation and investigation. TM: resources and project administration. MA, MG, MB, SS, MK, and ID: writing (review and editing). All authors have read and agreed to the published version of the manuscript.
